# Editorial: Advancing neonatal cardiology: a focus on precision medicine

**DOI:** 10.3389/fped.2025.1746122

**Published:** 2025-12-05

**Authors:** Ranjit Philip, Mark Weems, Sandeep Chilakala

**Affiliations:** 1 Department of Pediatrics, Le Bonheur Children’s Hospital, Memphis, TN, United States; 2 Department of Pediatrics, The University of Tennessee Health Science Center College of Medicine, Memphis, TN, United States

**Keywords:** CHD (congenital heart disease), patent ductus arteriosis, neonatolgy, Fetal & Placental pathology, echocadiography

## Introduction

Over the past decade, neonatal cardiology has undergone a remarkable transformation. The integration of precision medicine—anchored in genomics, advanced imaging, bioinformatics, and individualized physiology—has redefined how clinicians diagnose, prognosticate, and treat congenital and acquired cardiac conditions in the newborn period. This special issue, *Advancing Neonatal Cardiology: A Focus on Precision Medicine*, brings together original research and compelling case studies that exemplify this evolution—from molecular diagnosis to multimodal imaging and neurodevelopmental outcomes.

## Precision in diagnosis: from fetal life to early infancy

The articles in this issue underscore the value of precision diagnostics across the perinatal continuum. The case report, *Precise Prenatal Ultrasound Diagnosis of 22q11.2 Deletion Syndrome*, highlights how systematic fetal imaging protocols can guide early genetic investigation and anticipatory management, underscoring the expanding role of fetal cardiology within multidisciplinary precision medicine teams. Similarly, the *Fetal Total Anomalous Pulmonary Venous Connection: A Clinical Study of 31 Cases* reflects how detailed fetal echocardiographic assessment contributes not only to early diagnosis but also to postnatal surgical planning and improved outcomes.

Complementing these, the report on *Neonatal Right Atrial Mass of Uncertain Etiology* illustrates the diagnostic complexities encountered in the neonatal intensive care setting, where multimodal imaging and genetic insights intersect to refine clinical decisions in rare presentations.

## Integrating molecular and physiologic insights

Precision medicine extends beyond anatomic diagnosis to embrace molecular and physiologic variability. The paper, *The Gut Microbiota–Kawasaki Disease Axis: Emerging Evidence and Therapeutic Implications*, invites us to rethink inflammation-driven cardiac conditions through a microbiome–immune–vascular lens. As the field moves toward microbiota-targeted therapies, this perspective signals a paradigm shift toward understanding the neonatal and infant cardiac milieu as part of a larger systemic ecosystem.

The study *Dichotomy in Biventricular Function, Progress and Recovery among Extreme Preterm Infants with Persistent Ductus Arteriosus* demonstrates how serial echocardiographic assessment can reveal individualized cardiac trajectories, informing both timing of intervention and long-term follow-up strategies—an essential principle in neonatal precision cardiology.

## Linking the heart to the brain and beyond

The intersection of cardiac physiology, placental function, and neurodevelopment is a rapidly expanding frontier. The study *Placental Histology, Perioperative Brain Development, and Neurodevelopmental Outcome at One Year of Age in Patients Undergoing Neonatal Cardiac Surgery* explores the placental–cardiac–cerebral axis, suggesting that precision medicine in cardiology cannot exist in isolation from fetal and neurologic sciences. Understanding how placental pathology influences postoperative brain development opens new avenues for early risk stratification and neuroprotective strategies.

## Lessons from rare presentations and population trends

Rare case reports often illuminate fundamental physiologic principles. The report *Electrical Alternans of the Q–T Interval and Fatal Arrhythmias Caused by Neonatal Cardiac Tumor* reminds us of the vulnerability of the developing myocardium and the importance of vigilance in interpreting subtle electrocardiographic changes. At the other end of the spectrum, *Time Trends in Mortality of Congenital Heart Disease in Children Aged 0–14 Years* provides a macroscopic view of progress in care delivery, preventive strategies, and outcomes, emphasizing the collective success of multidisciplinary precision approaches across decades.

## Looking forward: the precision era in neonatal cardiology

Taken together, the studies in this issue reinforce that the future of neonatal cardiology lies in integration—of genomics with physiology, imaging with computation, and molecular insight with compassionate, individualized care. Precision medicine is not merely a technological advance; it is a framework that redefines how we understand and respond to variability among our smallest patients.

As this field advances, challenges remain—equitable access to advanced diagnostics, ethical considerations in genomic screening, and the translation of data into actionable care at the bedside. Yet, the contributions in this Research Topic offer optimism: they demonstrate how focused inquiry and collaboration continue to transform neonatal cardiology from a discipline of reactive care into one of proactive precision.

